# Sex, butterflies and molecular biology: when pigmentation met mimicry

**DOI:** 10.1111/pcmr.12260

**Published:** 2014-05-22

**Authors:** Richard H ffrench-Constant

Pigmentation, specifically melanism, is often the driving force behind classic examples of natural selection such as camouflage and mimicry. However, whilst pigmentation in insects has long been considered the exclusive preserve of the genetic model *Drosophila*, stunning new papers on the molecular basis of melanism and mimicry in butterflies are beginning to show how evolution can act directly on what has previously been viewed as strictly a developmental problem. For example, pigmentation and mimicry in the striking yellow, black and red ‘Postman’ butterflies of the genus *Heliconius* is controlled by transcription factors, such as *optix*, apparently redeployed from their roles in early development. Melanism is also important in sexual dimorphism and within butterflies is often used to generate mimicry-related sex-limited polymorphisms. Whilst a number of different butterfly families show such sex-limited mimicry, the swallowtail butterflies of the genus *Papilio* form an excellent model in which to study the genetics of pigmentation under strong natural selection. Clarke and Sheppard used swallowtails to study the genetics of mimicry and were the first to come up with the concept of a ‘supergene’, inferred to be a block of tightly linked genes somehow acting together to account for the seemingly complex array of different pigmentation phenotypes they observed in their genetic crosses.

Kunte et al. have now used a blistering series of genetic crosses, association mapping, transcriptome and genome sequencing to look at sex-limited melanism and mimicry in the Asian swallowtail *Papilio polytes,* which appears to be controlled by onesuch ‘supergene’. Here, males are black and yellow and are monomorphic and non-mimetic, whereas the females are either male like or belong to one of several different melanic morphs mimicking distasteful species from a different swallowtail genus, *Pachliopta*. Clarke and Sheppard had already shown that this female polymorphism, including the presence or absence of the swallow ‘tails’ themselves, was under the control of a single Mendelian locus or ‘supergene’. They envisaged that a tightly linked cluster of genes, each controlling different aspects of wing pattern, must be behaving as one single locus and that recombination within this supergene cluster would be unexpected or rare. Some 40 years later, Kunte and co-workers have confirmed that polymorphism is controlled by one single gene, *doublesex,* rather than a cluster of tightly linked loci. This helps explain why autosomal loci, such as the *H* locus that controls female-limited mimicry in *P. dardanus*, can be expressed in a sex-‘limited’ fashion rather than specifically being sex-*linked* as in the W (female specific chromosome)-linked, female-limited melanism in *P. glaucus*. This is of course because *doublesex* is responsible for *somatic* sex determination rather than the germline determined sex of the butterfly itself. Unfortunately, like any good piece of research, however, it also raises perhaps more questions than it answers. For example, *Drosophila doublesex* uses alternative splicing to control somatic sex differentiation, one splice form leading to male sexual differentiation and the other to female. But surprisingly, cloning of *doublesex* isoforms from mimetic and non-mimetic *P. polytes* males and females suggests no specific presence–absence correlation of isoforms with mimicry, rather gene expression levels themselves seem to be more important.

These findings highlight the huge gap now exposed between these regulatory ‘switch’ genes (*doublesex, optix* and *WntA*) and the genes controlling pigmentation itself. In swallowtails, yellow colour is formed by a pigment called papiliochrome and black is formed by melanin. In a strange twist, the precursor dopamine is required *both* to form the yellow papiliochrome (after its transformation to N-*β*-alanyl dopamine by N-*β*-alanyl dopamine synthase, the product of the *ebony* locus) *and* melanin itself (via dopamine quinone). To provide dopamine to the correct set of scales (yellow or black) at the correct stage in development, transcription of the dopa decarboxylase (DDC) encoding gene (which converts dihydroxy-phenylananine to dopamine) is therefore switched from the yellow pattern early in wing pigmentation to the black pattern just prior to butterfly eclosion (Koch et al., 1998). Such an exquisite spatial and temporal regulation of the enzymes involved in pigmentation is also mimicked by a similar regulation in which the scale cells themselves develop. In essence, therefore, pigmentation of the butterfly wing involves differential rates of development of differently coloured scales, in a process similar to developmental ‘heterochrony’. To become coloured (yellow or white), the scales must develop ‘early’, to be pigmented in the ‘papiliochrome window’, and in order to be black, they must delay their development to be exposed to the correct availability of melanin precursors (ffrench-Constant and Koch, 2003). So what has this all to do with *doublesex* and the control of somatic sex differentiation? Further what are the missing players in between the switch gene *doublesex* and the pigmentation gene *ddc*?

The final pigmentation of butterfly wings in the pupa, just before hatching, is set against a falling titre of the hatching hormone 20-hydroxyecdysone, and specific ecdysone receptor isoforms show colour pattern-specific expression during wing pattern development (Koch et al., 2003). The development of scale cells that are destined to be different colours must therefore be differentially triggered across the maturing butterfly wing, but again what is the critical trigger for the initiation of scale cell development itself? In this context, it is hard to understand how *doublesex*, a gene previously thought to be important in somatic sex determination, can also be deployed as a patterning gene. Thus, the *doublesex* switch not only appears to be involved in the genetic control of female morphs, but expression of the doublesex protein in developing scale cells also correlates with the yellowish white rays found on the forewing of mimetic females. Loehlin and Carroll, in their accompanying commentary in the same issue of *Nature* on pages 172–173 note that rather than simply indicating whether a specific tissue is male or female, *doublesex* expression is also elaborately regulated itself and shows tissue-specific expression in its *own right*. This means that *doublesex* is not only the female-limited switch gene but *also* a wing patterning gene.

Finally, if a tightly linked cluster of genes (a supergene) is indeed only *one* albeit rather complicated gene, what exactly is recombination within a supergene? Loehlin and Carroll already noted that complex genes like *doublesex* probably carry a significant number of regulatory elements themselves and that any ‘recombinants’ within this supergene (although none have been noted for *P. polytes* by either Clarke and Sheppard or indeed Kunte et al.), or others, may be recombination events between different *doublesex* enhancers, that is, specifically correspond to *recombinants within one gene rather than a set of clustered genes*. Further, Kunte et al. also found an inversion polymorphism with breakpoints flanking the *doublesex* gene; however, it is not clear how different inversions might lock together different *doublesex* regulatory elements to drive different patterns of scale cell development. These results are similar to inversions around the *P* locus of *Heliconius numata,* which control the wide range of mimetic morphs associated with Mullerian mimicry (Joron et al., 2011).

‘a tightly linked cluster of genes (a supergene) is indeed only one, albeit rather complicated, gene’

Significantly, many of these melanic mimicry examples, including *P. polytes* and *H. numata*, show dominance series amongst the different mimetic alleles with more melanic morphs being dominant over paler ones. If we now understand these to be alleles of a single gene (*doublesex* or *P*), then it may be easier to understand how such dominance series have evolved. Different inversions may therefore simply ‘lock together’ different enhancer elements and thus drive morph specific patterns of expression from different alleles of a single switch gene. This may also help to explain the famous ‘industrial’ melanism of the peppered moth, *Biston betularia*, where again darker forms are dominant over lighter ones and again melanism looks to be under control of a single locus at the equivalent chromosomal location to *H. numata P* (Van't Hof et al., 2011). This very brief review of pigmentation and melanism in butterflies and moths therefore gives us two important ‘take-home messages’. First, pigmentation is about *more* than just ‘development’ and can in fact act as a major driver of natural selection. Second, and as a direct correlate to this, understanding the developmental heterochrony that underlies insect pigmentation is critical to our understanding of the molecular basis of natural selection itself.
